# Virtual Reality–Enhanced Training for Trauma-Informed Care Among Residential and Child Mental Health Professionals: Pre-Post Evaluation Study

**DOI:** 10.2196/86543

**Published:** 2026-04-17

**Authors:** Kostadin Kostadinov, John Goodwin, Gunter Groen, Astrid Jörns‑Presentati, Sinéad Heffernan, Áine O’Donovan, Maria O’Malley, Ryan Biskupovic Goulding, James O’Mahony, Stephanie Allen, Margaret Curtin, Satu Haapalainen, Jami Aho, Gergana Petrova, Svetla Ivanova, Valentina Lalova, Joonas Korhonen

**Affiliations:** 1 Department of Social Medicine and Public Health, Faculty of Public Health, Medical University of Plovdiv, Bul. Vasil Aprilov 15A, Plovdiv, 4000, Bulgaria, 359 0897065910; 2 School of Nursing & Midwifery, University College Cork, Cork, Ireland; 3 Department of Social Work, Hamburg University of Applied Sciences (HAW Hamburg), Hamburg, Germany; 4 West Cork Women Against Violence Project CLG, West Cork Beacon, Cork, Ireland; 5 School of Nursing and Midwifery, Trinity College Dublin, Dublin, Ireland; 6 Health and Well-Being, Turku University of Applied Science, Turku, Finland; 7 Turku University of Applied Sciences, Turku, Finland; 8 Department of Nursing Care, Faculty of Public Health, Medical University of Plovdiv, Plovdiv, Bulgaria

**Keywords:** trauma-informed care, virtual reality, professional education, child welfare, residential care

## Abstract

**Background:**

Trauma-informed care (TIC) is a framework that embeds safety, trust, choice, collaboration, and empowerment into practice. Although training improves TIC attitudes, implementation in European residential child and adolescent settings remains inconsistent, and immersive technologies such as virtual reality (VR) are underevaluated for this purpose.

**Objective:**

This study aims to evaluate whether a VR-enhanced training program improves TIC attitudes among staff and trainees in residential child and adolescent care across European sites and to examine site-level heterogeneity.

**Methods:**

We conducted a multisite pre-post evaluation within the European Union–co-funded Safe4Child project. Participants completed a standardized online TIC module followed by a mentor-facilitated VR simulation. Attitudes were measured using the ARTIC-10 (10-item version of the Attitudes Related to Trauma-Informed Care; 7-point) scale immediately before the online module and immediately after the VR simulation; the design, therefore, evaluates the combined program rather than VR in isolation. Analyses included Wilcoxon signed-rank tests, multivariable regression, and Bayesian models with skeptical priors.

**Results:**

Among 79 matched participants from Bulgaria, Finland, and Germany, mean (SD) ARTIC-10 scores increased from 5.33 (1.05) to 5.57 (1.20; *P*<.001; Cohen *d*=0.22). Effects varied by site. Germany showed significant improvement (Cohen *d*=0.56), whereas Bulgaria and Finland did not reach significance. Bayesian estimation yielded a mean change of 0.24 points (95% CI 0.11‐0.37) with *P* (*Δ*>0) ≈ 1.00. Prior trauma-related training showed a probable but uncertain additional benefit.

**Conclusions:**

The VR-enhanced TIC program produced a small overall improvement in attitudes, with a moderate effect in Germany, suggesting that immersive training can strengthen TIC learning when aligned with local contexts. The absence of a control group precludes attributing effects specifically to VR. Larger comparative trials are needed to determine whether VR confers advantages over conventional training approaches.

## Introduction

### Background

Trauma-informed care (TIC) is recognized as an organizational and clinical framework that acknowledges the widespread prevalence and significant impact of trauma, especially among vulnerable groups such as children and adolescents in residential care settings [1]. Core principles including safety, trustworthiness, choice, collaboration, and empowerment are integrated into routine practices through this approach, which acknowledges the link between intergenerational and childhood trauma to wellness and therefore represents a fundamental shift from inquiring, “What is wrong with you?” to “What happened to you?” [2]. Within residential programs, TIC has been associated with notable outcomes, including reductions in behavioral incidents, enhanced placement stability, and improved relationships between carers and children [3]. Furthermore, the adoption of TIC principles has been linked to decreased reliance on restrictive practices, such as seclusion and restraint, which are often used in managing behaviors that challenge but can exacerbate trauma responses if not handled sensitively [4]. TIC not only mitigates immediate risks but also fosters long-term resilience by addressing underlying trauma histories, potentially reducing the cycle of retraumatization in residential environments [5].

### Challenges in Implementing TIC

Despite its established benefits, the implementation of TIC is hindered by various barriers. Organizational resistance to cultural shifts, insufficient staff training and awareness, burnout among personnel, and systemic limitations such as funding shortages or rigid policies have been identified in systematic reviews as key obstacles [6,7]. These challenges often create a gap between what policies promise and what happens in practice. To close this gap, targeted strategies are needed. In Europe, there are additional barriers, such as differences between national health care systems, language and cultural diversity, and varying regulations, all of which can make it harder to apply TIC in the same way everywhere [8]. For instance, in European Union member states, resource allocation disparities and a lack of interdisciplinary collaboration have been noted as exacerbating factors, particularly in residential settings serving diverse migrant populations where trauma experiences may intersect with cultural stigma or access inequities [9]. These barriers underscore the importance of viewing TIC implementation as a policy shift, where overcoming them requires not only training but also structural reforms to align incentives. For example, progress in TIC could be tracked with specific measures that are linked to how residential facilities receive funding or how staff performance is evaluated [7].

### The Role of Training in TIC Adoption

Training is regarded as essential for TIC adoption [10], given that staff attitudes and beliefs significantly influence practice efficacy. Such training is crucial for aligning daily practices with TIC principles, thereby addressing the policy-practice disconnect [11]. Effective training should incorporate experiential elements to challenge implicit biases, particularly in managing behaviors that challenge where reactive responses might prevail without adequate preparation [12]. In child and adolescent settings, this could extend to scenario-based learning that simulates real-world interactions, promoting proactive rather than punitive approaches [13].

### Immersive Technologies and Virtual Reality

Substantial progress has been made in recent years regarding immersive technologies in health care [14], with specific applications in mental health. Augmented reality, for example, has been used effectively for assessing and treating contamination-based obsessive-compulsive disorder [15], and haptic feedback has been explored as a novel method for anxiety management [16]. A considerable body of literature focuses on virtual reality (VR), which has demonstrated efficacy in alleviating suicidal ideation [17], reducing symptoms of depression, anxiety, and stress [18], and improving attitudes toward schizophrenia [19]. VR’s role in health care education and behavioral training is supported by evidence indicating its capacity to enhance empathy and perspective-taking through simulations [20,21]. Studies have shown that VR simulations for health care students foster empathy for individuals experiencing psychosis or schizophrenia [22,23], and improvements in empathy domains among medical students have been noted [24]. Although VR applications for trauma survivors exist [25,26], the use of VR in educating staff about TIC remains underexplored.

VR provides distinct advantages for TIC training, such as promoting empathy, facilitating perspective-taking, and enabling experiential learning via realistic scenarios [27]. Its cost-effectiveness, repeatability, and standardization have been highlighted in scoping reviews of medical education [28], positioning it as a viable tool for TIC. Emerging applications include immersive simulations that deepen understanding of TIC principles and enhance application skills [29].

This study underscores the potential of VR to safely recreate trauma-informed environments, allowing staff to practice de-escalation without real-world risks. This is particularly important for managing risk behaviors in children, which is a key factor in specialized childcare [30]. Recent advancements, such as VR programs for trauma-informed de-escalation in hospitals and simulations for understanding adverse childhood experiences, illustrate how VR can transform training by immersing users in scenarios that highlight the impact of space design or behavioral triggers on trauma responses [31,32]. In residential settings, VR could further address aggressive behaviors by enabling trainee teachers or carers to rehearse interventions in virtual classrooms or homes, potentially reducing restrictive practices through better-prepared staff [33].

### The Need for Evaluation in European Residential Settings

Given the barriers to TIC implementation and the potential of VR, there is a critical need to develop and evaluate VR-enhanced TIC training in diverse care environments, including residential child and adolescent settings across multiple European contexts. This study addresses this gap through a multicenter pilot conducted in residential facilities and a child psychiatric ward across three European Union countries.

The primary objective of this study was to examine whether participation in a VR-based training program leads to changes in attitudes toward TIC among staff and trainees in residential child and adolescent care settings. Secondary objectives included exploring variability in outcomes across different national contexts and mentor teams, as well as examining participant characteristics associated with attitudes toward TIC.

## Methods

This evaluation is reported in line with the TREND (Transparent Reporting of Evaluations with Non-Randomized Designs) statement (Checklist 1) [34], where applicable, and the intervention is described using TIDieR (Template for Intervention Description and Replication; Checklist 2) [35].

### Study Design

A multisite pre-post evaluation design was used to evaluate the short-term impact of a VR-enhanced training program on professionals’ attitudes toward TIC. This within-subject design enabled each participant to serve as their own control, minimizing confounding due to interindividual variability and enhancing sensitivity to changes in attitudes [36].

### Safe4Child Project

This study was conducted as part of the European Union–co-funded project Safe4Child—Caring Violent Child Safely in Child Psychiatric and Residential Units [37]. The project aimed to strengthen frontline workers’ competencies in managing behaviors that challenge in children’s residential and psychiatric care settings across four European countries (Finland, Germany, Bulgaria, and Ireland). A VR environment and an online continuing education course were developed to support this goal. The project addressed the underlying causes of challenging behaviors and specifically sought to reduce the use of restrictive practices while promoting trauma-informed and child-centered approaches. It also aimed to enhance staff resilience and well-being, improve safety for both children and professionals, and foster more sustainable and ethically grounded practices. All materials produced within the project are open access and available to health and social care professionals on the Safe4Child website.

### Setting and Participants

The study was initiated across 4 European Union countries in collaboration with Hamburg University of Applied Sciences (HAW, Germany), Turku University of Applied Sciences (TUAS, Finland), University College Cork (Ireland), and the Medical University of Plovdiv (MUP, Bulgaria). Participants were recruited through convenience sampling coordinated by local research partners within the Safe4Child consortium. However, the postintervention assessment was completed only at 3 sites—Germany, Finland, and Bulgaria. As a result, data from University College Cork comprised preintervention measurements only and were excluded from pre-post change analyses; all inferential results therefore reflect the 3 sites with matched observations.

Eligible participants included professionals and trainees in health care and social work currently employed in, or preparing for, roles in residential and child and adolescent care settings or child psychiatric wards. A total of 120 participants were enrolled, with 30 from each country, consistent with the pilot phase requirements of the Safe4Child grant agreement. Across study sites, participants represented both trainees and practicing professionals with varying degrees of prior exposure to child welfare and mental health care. In Germany, the sample consisted of undergraduate students in social work at HAW, most of whom had completed field placements, primarily in child welfare settings, though only a small subset had professional roles in residential childcare. In Finland and Bulgaria, the cohorts included a broader mix of professionals and trainees working in or preparing for roles in child welfare and mental health contexts.

Participation was voluntary, and informed consent was obtained in accordance with ethical standards and institutional review board approvals in each country.

### Intervention

The intervention comprised 2 sequential components designed to build foundational knowledge and practical skills in TIC. The first component was a standardized, self-paced online training module (5 ECTS), delivered in English, covering TIC principles, including the neurobiological effects of trauma, relational dynamics in care, and strategies for managing distress and behavioral escalation in residential and psychiatric settings. Interactive self-evaluation quizzes were embedded to reinforce learning, with the module intended for completion within 2 weeks.

Following the online modules, participants engaged in a VR simulation, delivered individually and uniformly across all study sites. The 15 to 25-minute session was mentor-facilitated and used a standardized scenario depicting a distressed child in a residential care setting; full intervention details are provided in Multimedia Appendix 1 and are documented using the TIDieR checklist [35].

The scenario and mentoring protocol were not culturally customized but were developed to be universally applicable, informed by thematic analysis of case descriptions and semistructured interviews with frontline professionals across the 3 countries [38,39]. This ensured the scenario reflected common patterns of behavioral escalation and staff-child interactions, remaining relevant across national contexts. Mentors provided real-time guidance, and each session concluded with a structured debriefing to promote critical reflection and reinforce TIC approaches.

### Outcome Measures

The primary outcome was the shift in participants’ attitudes toward TIC, assessed using the ARTIC-10 (10-item version of the Attitudes Related to Trauma-Informed Care) scale [40]. The ARTIC scale, comprising 45 items, is commonly used to evaluate these attitudes, while its 10-item abbreviated version exhibits robust internal consistency (Cronbach *α*=0.89) and responsiveness to training-induced changes [40]. Improvements in ARTIC scores posttraining reflect enhanced trauma-informed perspectives among staff [41-43]. This validated instrument, designed for rapid administration, maintains strong psychometric properties [44,45]. Responses were captured on a 7-point Likert scale, with composite scores calculated by averaging all items after reverse-scoring items 2, 4, 6, 8, and 9, per standard procedures. Higher scores indicate stronger alignment with TIC principles. Although the ARTIC-10 demonstrates acceptable internal consistency and responsiveness to training, recent psychometric evaluations have identified limitations regarding factor structure stability and cultural invariance across diverse populations [46]. These properties warrant caution when comparing scores across national or cultural contexts. The ARTIC-10 was administered at 2 time points via a secure, General Data Protection Regulation (GDPR)-compliant, self-administered online survey system: immediately before commencement of the online module (preintervention) and immediately after completion of the VR simulation (postintervention). Data collection occurred between January 2024 and March 2024. This design evaluates the effect of the combined training program (online module plus VR); however, the contribution of the VR component cannot be isolated from that of the preceding didactic content.

### Data Analysis

All analyses were performed using R version 4.5.1 (R Foundation for Statistical Computing). Descriptive statistics summarized participant demographics and ARTIC-10 scores. Continuous variables were reported as means and SDs for normally distributed data or medians and IQRs for non-normally distributed data. Categorical variables were expressed as frequencies and percentages. Computational reproducibility materials, including data-processing scripts, model code, and figure-generation routines, are provided in Multimedia Appendix 2 (Code Reproduction).

Only participants with complete, matched preintervention and postintervention responses were included (N=79). Matching was achieved using anonymized identifiers assigned by local investigators to ensure within-subject comparability. To assess measurement reliability, we evaluated the internal consistency of the ARTIC-10 at preintervention and postintervention, overall and stratified by country. The Cronbach α was estimated from the 10-item responses, with its asymptotic SE, and Wald-type 95% CIs.

To evaluate the intervention’s effect on TIC attitudes, a multilayered analytical approach was adopted. The Wilcoxon signed-rank test was used to assess within-subject differences in ARTIC-10 scores, accounting for non-normal distribution. A binomial test evaluated the proportion of participants with increased ARTIC-10 scores postintervention, using a 50% threshold for significance. Effect size was estimated using Cohen *d*, with thresholds of 0.2, 0.5, and 0.8 for small, medium, and large effects, respectively.

To explore contextual and demographic influences, multivariable regression modeling was applied. Linear regression modeled the ARTIC-10 score difference (score_diff = artic_post - artic_pre), and logistic regression modeled the probability of an increase (increase_fct, postintervention > preintervention). Predictors included country, sex, age, prior health care experience, years of experience with children and adolescents, education level, previous trauma-related training, and type of employing organization. Data on field placement duration and specific focus on child welfare were collected only for the German cohort, as these variables were relevant to participants’ undergraduate Social Work curriculum at HAW; equivalent structured placement data were not systematically recorded at the Finnish and Bulgarian sites. These predictors were therefore examined in exploratory site-specific analyses for Germany only, and results should be interpreted accordingly. A 2-sided significance level of *P*<.05 was used for all frequentist tests.

In addition to the prespecified frequentist tests, we used a Bayesian framework to quantify the magnitude and uncertainty of pre–post change and to model site-level heterogeneity under explicit priors. For each participant with matched observations (N=79), the paired change was computed as $\Delta_{i}\text{=}{\text{ARTIC}}_{\text{post},i}\text{-}{\text{ARTIC}}_{\text{pre},i}$ after standard scoring.

We first fit an intercept-only model:


$$\Delta_{i}∼\text{Student-}t\left(\mu ,\sigma ,\nu \right),\mu \text{=}\alpha ,$$

with a skeptical prior α∼N(0,0.3) to encode the expectation of little to no average change on the 1‐7 ARTIC scale, and a robust residual prior $\sigma ∼t_{3}\left(0,0.7\right)$ to mitigate the influence of outliers. The 2-tailed Student *t* likelihood provides additional robustness to occasional extreme differences.

To examine contextual moderation, we then specified:


$$\Delta_{i}∼\text{Student-}t\left(\mu_{i},\sigma ,\nu \right),\mu_{i}\text{=}\alpha \text{+}\beta_{\text{country}\left[i\right]}\text{+}\gamma 1\left\{{\text{prior training}}_{i}\text{=}\text{Yes}\right\}$$

treating country as a factor with Bulgaria as the reference (default alphabetical ordering in R; the choice of reference category does not affect pairwise contrasts or substantive interpretation) and adding an indicator for prior trauma-related training. Regression coefficients received weakly informative priors β,γ∼N(0,0.3); the same priors on σ and ν and the same likelihood as above were used.

Models were fit in Bayesian regression models using Stan (Stan Development Team) [47] (Hamiltonian Monte Carlo via Stan) with 4 chains, 4000 iterations per chain (1000 warm-up), adapt_delta 0.98‐0.99, and maximum treedepth 12. Convergence was assessed using $\hat{R}\approx 1.00$ and large effective sample sizes, and model adequacy was inspected with posterior predictive checks.

Inference focused on posterior means, 95% credible intervals (CrI), and probabilities for decision-relevant events. Specifically, we report P(Δ\gt 0) as the probability of any improvement, P(Δ\gt 0.20) as a benchmark for a practically larger lift, and the share of posterior mass within a region of practical equivalence (ROPE). The ROPE formalizes “negligible” change: we prespecified ±0.10 ARTIC points, which is approximately 1.4% of the total scale range and smaller than the observed within-person dispersion, making it a conservative threshold. When most posterior mass lies inside the ROPE, we interpret the effect as practically null; when most mass lies above both zero and the ROPE (and, where relevant, beyond +0.20), we interpret it as a practically meaningful improvement. Country-specific posteriors and contrasts were obtained by transforming the linear predictor, propagating full uncertainty from all parameters. Reproducible code for all Bayesian analyses is provided in Multimedia Appendix 2.

### Ethical Considerations

This study was conducted in accordance with the ethical principles established in the Declaration of Helsinki (World Medical Association, 2013) [48]. Ethical approval was granted by the Human Sciences Ethics Committee of Turku University of Applied Sciences (Statement LP06_2022, dated September 5, 2022), covering all participating study sites. All participants received a comprehensive information sheet describing the study objectives, procedures, voluntary nature of participation, and their right to withdraw at any time without consequence. Written informed consent was obtained from all participants prior to data collection.

All data were collected, processed, and stored in accordance with the GDPR (Regulation [EU] 2016/679). A GDPR-compliant privacy notice was provided to all participants alongside the study information materials. Participant confidentiality was maintained throughout; data were anonymized prior to analysis and no identifying information is reported in this manuscript. Participants received no financial compensation for their participation in this study.

## Results

### Participant Characteristics

Only participants who completed both the preintervention and postintervention questionnaires were included in the analysis, reducing the final analytical sample from the original 120 respondents to 79. The overall response rate was estimated at 66%. The sample was predominantly female (n=71, 90%), with the highest proportion of male participants coming from Finland and Germany (13% each, Table 1).

**Table 1.** Participant characteristics by country (N=79).

Characteristic	Overall (N=79)	Bulgaria (n=25)	Finland (n=24)	Germany (n=30)
Sex (male), n/N (%)	8/79 (10)	1/25 (4)	3/24 (13)	4/30 (13)
Age (y), mean (SD)	29.8 (10.7)	34.1 (14.5)	26.3 (6.0)	29.1 (8.9)
Education^a^, n/N (%)				
Student	58/79 (73)	12/25 (48)	21/24 (88)	25/30 (83)
Diploma	3/79 (3.8)	2/25 (8)	—^b^	1/30 (3.3)
Bachelor’s degree	6/79 (7.6)	—	3/24 (13)	3/30 (10)
Master’s degree	7/79 (8.9)	6/25 (24)	—	1/30 (3.3)
Doctorate	5/79 (6.3)	5/25 (20)	—	—
Experience				
Health care experience (y), mean (SD)	6.7 (8.9)	12.6 (13.5)	3.8 (3.3)	3.9 (2.9)
Specialized experience (y), mean (SD)^a^	3.6 (4.8)	4.6 (6.9)	1.7 (2.7)	4.0 (3.3)
No working experience, n/N (%)	11/79 (14)	3/25 (12)	6/24 (25)	2/30 (6.7)
Previous TIC^c^ training (yes), n/N (%)	17/79 (22)	9/25 (36)	4/24 (17)	4/30 (13)
Type of organization^a^				
None	62/79 (78)	24/25 (96)	18/24 (75)	20/30 (67)
Child welfare (residential)	8/79 (10)	—	1/24 (4.2)	7/30 (23)
Mental health	8/79 (10)	—	5/24 (21)	3/30 (10)
Pediatric clinics or units	1/79 (1.3)	1/25 (4)	—	—

a Indicates statistically significant differences between sites (*P*<.05).

b Not applicable.

c TIC: trauma-informed care.

The mean age of participants was 29.8 (SD 10.7) years, with Bulgarian participants being older on average (34.1, SD 14.5 y) compared to those from Finland (26.3, SD 6.0 y) and Germany (29.1, SD 8.9 y). Educational backgrounds varied, with 73% of participants identifying as students. Among countries, Finland (88%) and Germany (83%) had the highest proportions of student participants, while Bulgaria showed a more diverse distribution, including individuals with doctoral degrees (20%) and master’s degrees (24%).

On average, participants had 6.7 (SD 8.9) years of health care experience, with Bulgarian participants reporting the most extensive experience (mean 12.6, SD 13.5 y). Specialized experience averaged 3.6 (SD 4.8) years across all participants. Between-site comparisons revealed statistically significant differences in educational background (*χ*²_8_=28.66; *P*<.001), type of employing organization (*χ*²_6_=17.50; *P*=.008), and years of experience working with children and adolescents (Kruskal-Wallis *χ*²_2_=8.23; *P*=.016). No significant differences were observed for sex (*χ*²_2_=1.52; *P*=.47), age (Kruskal-Wallis *χ*²_2_=1.20; *P*=.55), years of health care experience (Kruskal-Wallis *χ*²_2_=1.06; *P*=.59), or prior trauma-related training (Fisher exact test, *P*=.13). These baseline differences reflect the heterogeneous composition of cohorts across sites—predominantly undergraduate students in Germany, with more diverse professional backgrounds in Bulgaria and Finland—and were accounted for in multivariable regression models.

Overall, 22% of participants reported previous training related to TIC, with the highest prevalence among Bulgarian participants (36%). The majority of participants (78%) reported no affiliation with specific organizational types, though a smaller subset identified with child welfare or mental health organizations. Notably, 23% of German participants were affiliated with child welfare organizations, while 21% of Finnish participants worked in mental health settings.

### Internal Consistency (Cronbach α) of the ARTIC-10

Internal consistency of the ARTIC-10 was good at baseline and improved after the intervention. Overall, Cronbach α increased from 0.839 (95% CI 0.792‐0.886) preintervention to 0.894 (95% CI 0.863‐0.926) postintervention. By country, reliability was acceptable to good at baseline: Bulgaria 0.703 (95% CI 0.578‐0.827), Finland 0.810 (95% CI 0.703‐0.918), and Germany 0.809 (95% CI 0.708‐0.910)—and generally stable or higher postintervention: Bulgaria 0.836 (95% CI 0.761‐0.911), Finland 0.843 (95% CI 0.740‐0.946). Germany showed a modest decrease to 0.765 (95% CI 0.621‐0.909) postintervention, remaining within an acceptable range for group comparisons.

These values indicate that the instrument demonstrated adequate internal consistency across sites and time points, with the strongest reliability observed in the pooled postintervention data.

### Changes in ARTIC-10 Scores Following the Intervention

Analysis of ARTIC-10 scores revealed a significant overall improvement in TIC attitudes following the intervention. The mean score increased from 5.33 (SD 1.05; range 2.3‐7.0) at baseline to 5.57 (SD 1.20; range 2.1‐7.0) postintervention. A Wilcoxon signed-rank test confirmed that this change was statistically significant (*P*<.001), with a small-to-moderate effect size (Cohen *d*=0.22).

To explore potential heterogeneity in outcomes across implementation contexts, we conducted center-specific analyses. A significant improvement in ARTIC-10 scores was observed among participants in Germany (HAW; *P*<.001), with a moderate effect size (Cohen *d*=0.56). In contrast, no significant changes were detected among participants from Bulgaria (MUP; *P*=.796) or Finland (TUAS; *P*=.19).

**Figure 1.**
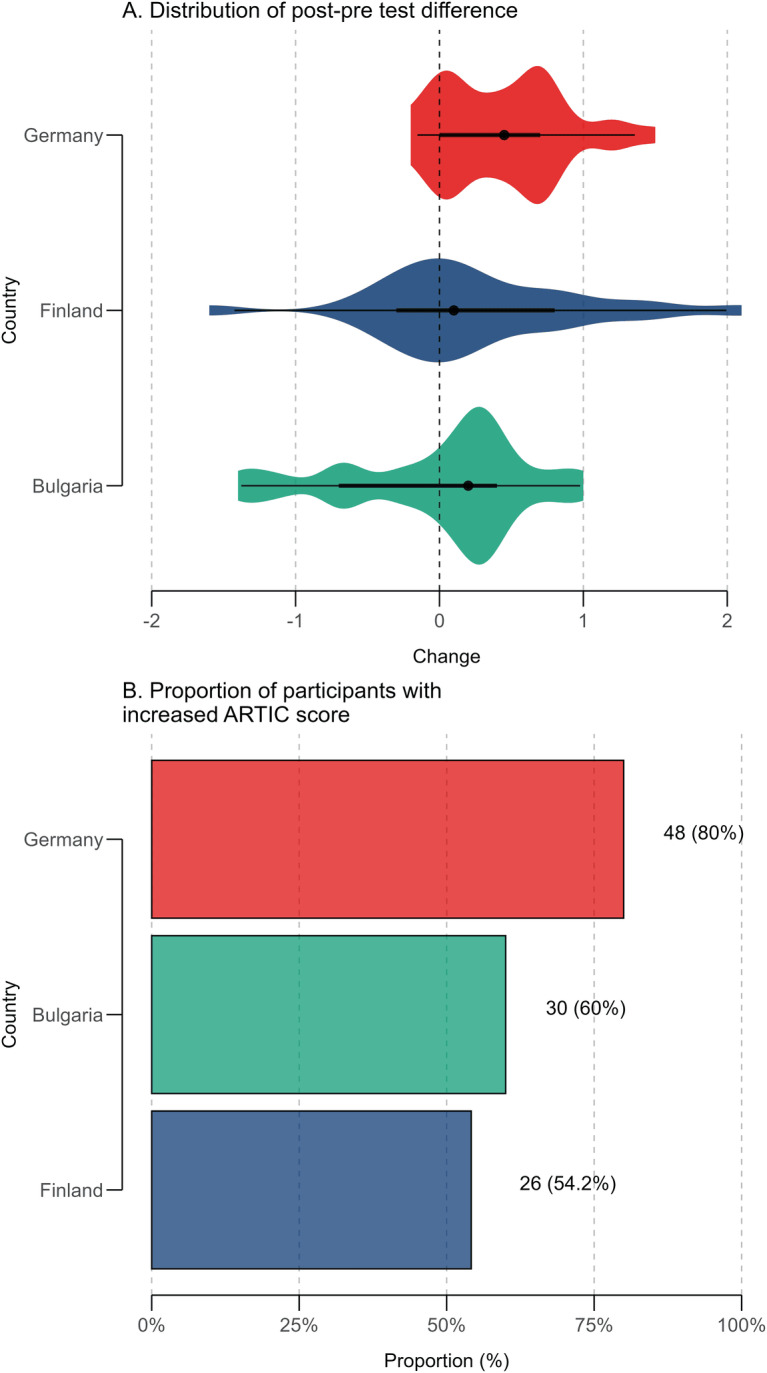
Changes in ARTIC-10 (10-item version of the Attitudes Related to Trauma-Informed Care) scores by country. Panel (A) displays the distribution of change scores (post–pre intervention) in ARTIC-10 for each country. Eye plots show the density of individual scores, with the mean and 95% CIs indicated at the center. Panel (B) presents the proportion of participants in each country who demonstrated an increase in ARTIC-10 scores following the intervention, also with 95% CIs. In both panels, countries are color-coded for visual distinction.

The proportion of participants exhibiting an increase in ARTIC-10 scores (defined as postintervention > preintervention) also varied by country. In Germany, 80% of participants demonstrated improvement (95% CI 61.4%‐92.3%), compared with 60% in Bulgaria (95% CI 38.7%‐78.9%) and 54.2% in Finland (95% CI 32.8%‐74.4%; Figure 1).

To further examine differences in outcomes, participants were stratified based on the direction of change in their ARTIC-10 scores: those with an increase (*Δ*>0) and those with a decrease or no change (Table 2). The increase group reported significantly higher postintervention scores (mean 5.9, SD 0.9) compared to the decrease group (mean 5.0, SD 1.4; *P*=.005). The average relative change in ARTIC-10 scores across the full sample was 4.9% (SD 14.1%). The increase group experienced a mean improvement of 11.6% (SD 10.6%), while the decrease group exhibited a mean reduction of −8.0% (SD 10.6%; *P*<.001).

**Table 2.** Descriptive statistics of participants by ARTIC-10 (10-item version of the Attitudes Related to Trauma-Informed Care) score change group.

Characteristic	Overall (N=79)	Decrease (n=27)	Increase (n=52)	*P* value
ARTIC-10 scores, mean (SD)				
Preintervention	5.3 (1.1)	5.4 (1.2)	5.3 (1.0)	.70
Postintervention	5.6 (1.2)	5.0 (1.4)	5.9 (0.9)	.005^a^
Relative change (%)	4.9 (14.1)	−8.0 (10.6)	11.6 (10.6)	<.001^b^
Country, n/N (%)				
Bulgaria	25/79 (32)	10/27 (37)	15/52 (29)	.50
Finland	24/79 (30)	11/27 (41)	13/52 (25)	.15
Germany	30/79 (38)	6/27 (22)	24/52 (46)	.04^c^
Type of organization, n/N (%)				
None	62/79 (78)	21/27 (78)	41/52 (79)	>.90
Child welfare (residential)	8/79 (10)	3/27 (11)	5/52 (9.6)	>.90
Mental health	8/79 (10)	3/27 (11)	5/52 (9.6)	>.90
Pediatric	1/79 (1.3)	0/27 (0)	1/52 (1.9)	>.90
Demographics and experience				
Sex (male), n/N (%)	8/79 (10)	4/27 (15)	4/52 (7.7)	.40
Age (y), mean (SD)	29.8 (10.7)	31.9 (11.4)	28.8 (10.3)	.20
Health care experience (y), mean (SD)	6.7 (8.9)	8.2 (8.4)	5.9 (9.2)	.07
No prior experience, n/N (%)	11/79 (14)	5/27 (19)	6/52 (12)	.50
Prior TIC^d^ training, n/N (%)	17/79 (22)	4/27 (15)	13/52 (25)	.30

a *P*<.01.

b *P*<.001.

c *P*<.05.

d TIC: trauma-informed care.

A significantly higher proportion of participants in the increase group were from Germany (46% vs 22% in the decrease group; *P*=.04). No significant differences were observed by country of origin for Bulgaria (*P*=.50) or Finland (*P*=.15). Prior trauma-related training was more common in the increase group (25% vs 15%), although the difference was not statistically significant (*P*=.30). Similarly, the two groups did not differ significantly by sex, age, or organization type.

Participants in the decrease group tended to be slightly older (mean 31.9 y, SD 11.4 y) than those in the increase group (mean 28.8, SD 10.3 y), though this difference did not reach statistical significance (*P*=.20). Those in the decrease group also reported more years of health care experience (mean 8.2, SD 8.4 y) compared to the increase group (mean 5.9, SD 9.2 y; *P*=.07). Prior health care experience was marginally more common in the decrease group (19% vs 12%; *P*=.50).

### Multivariable Regression Analysis

**Figure 2.**
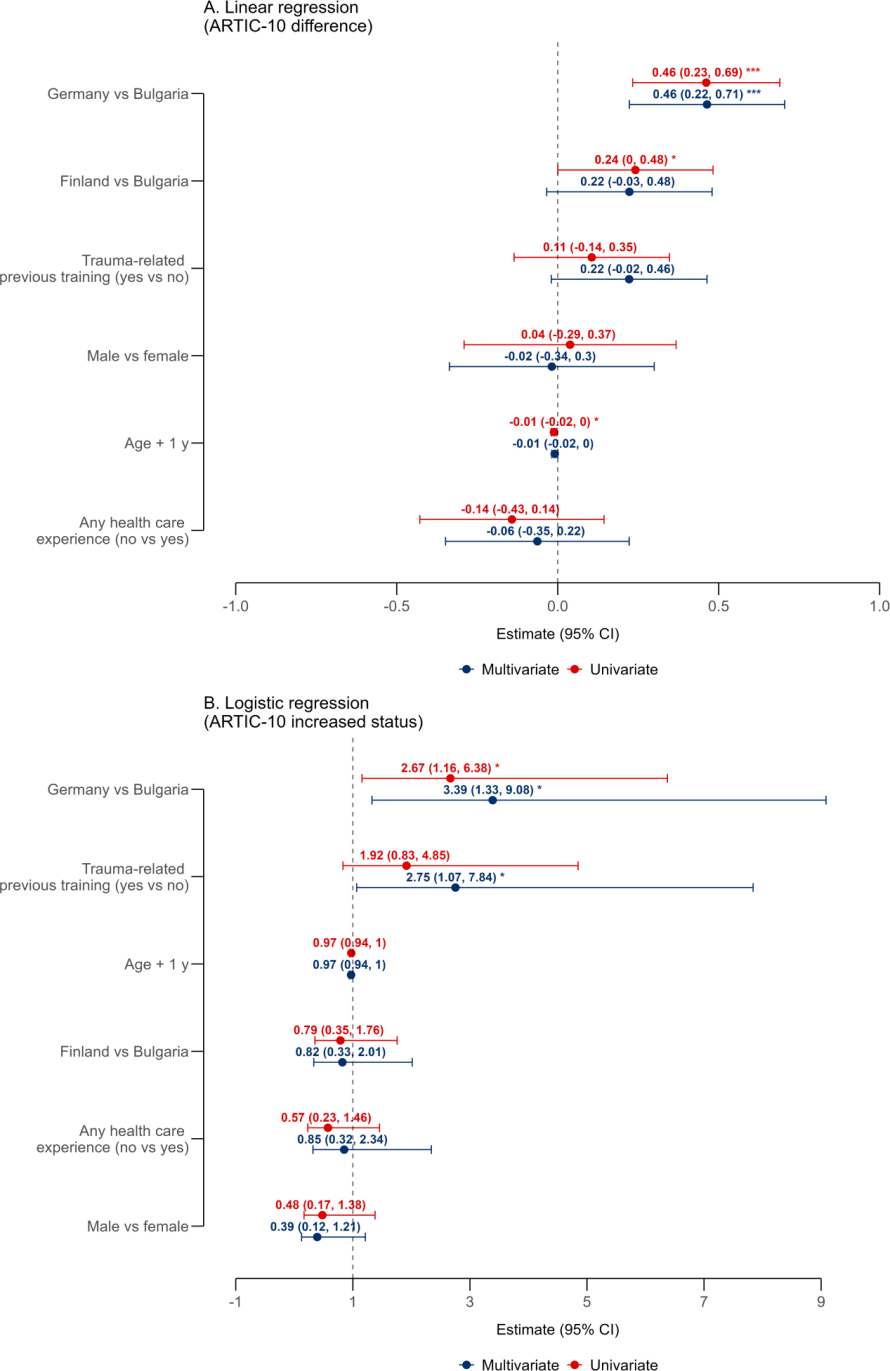
Predictors of change in ARTIC-10 (10-item version of the Attitudes Related to Trauma-Informed Care) scores. (A) Linear regression results shown as a forest plot of β coefficients with 95% CIs. Red bars represent univariate estimates; blue bars indicate multivariable model estimates. The vertical dashed line at 0 denotes the null effect. (B) Logistic regression results displayed as odds ratios (ORs) with 95% CIs for the likelihood of a positive change in ARTIC-10 scores. The vertical dashed line at 1 represents the null value (no effect). Bulgaria serves as the reference category for country comparisons. Asterisks indicate statistical significance: *P<.05; ***P<.001.

A linear regression model was used to identify predictors of change in ARTIC-10 scores, defined as the difference between post- and preintervention scores. As shown in Figure 2, the univariate analysis revealed that participants from HAW (Germany) experienced a significantly greater improvement in ARTIC-10 scores compared to those from MUP (Bulgaria), which served as the reference category (*β*=0.461, 95% CI 0.132‐0.790; *P*=.007). The effect for TUAS, Finland, also compared to Bulgaria, was not statistically significant (*β*=0.241, 95% CI −0.106 to 0.589; *P*=.170). Younger age was marginally associated with greater score improvements (*β*=−0.012, 95% CI −0.025 to 0.002; *P*=.083), suggesting that younger participants tended to benefit more from the intervention.

In the multivariable linear regression, the effect of the program delivery center at HAW, Germany, remained strong and statistically significant (*β*=0.464, 95% CI 0.111‐0.816; *P*=.01), while the association with age was not significant (*β*=−0.009, 95% CI −0.023 to 0.004; *P*=.18). These findings indicate that country-level differences in score change were not fully accounted for by individual-level covariates, such as age or prior experience.

To examine the likelihood of any positive change in ARTIC-10 scores, a multivariable logistic regression model was fitted. As depicted in Figure 2, both the center delivering the intervention and prior trauma-related training emerged as predictors. Participants at HAW, Germany, showed a trend toward higher odds of positive change compared to those in Bulgaria, though this did not reach statistical significance (odds ratio 3.39, 95% CI 0.90‐14.00; *P*=.077). Similarly, participants who had received previous trauma-related training showed a nonsignificant tendency toward a greater likelihood of improvement (odds ratio 2.75, 95% CI 0.73‐12.60; *P*=.16). While neither association attained conventional significance at *α*=.05, the direction and magnitude of effects suggest potential roles for site-related factors and foundational knowledge in facilitating intervention responsiveness, warranting further investigation in adequately powered studies.

### Bayesian Analysis

**Figure 3.**
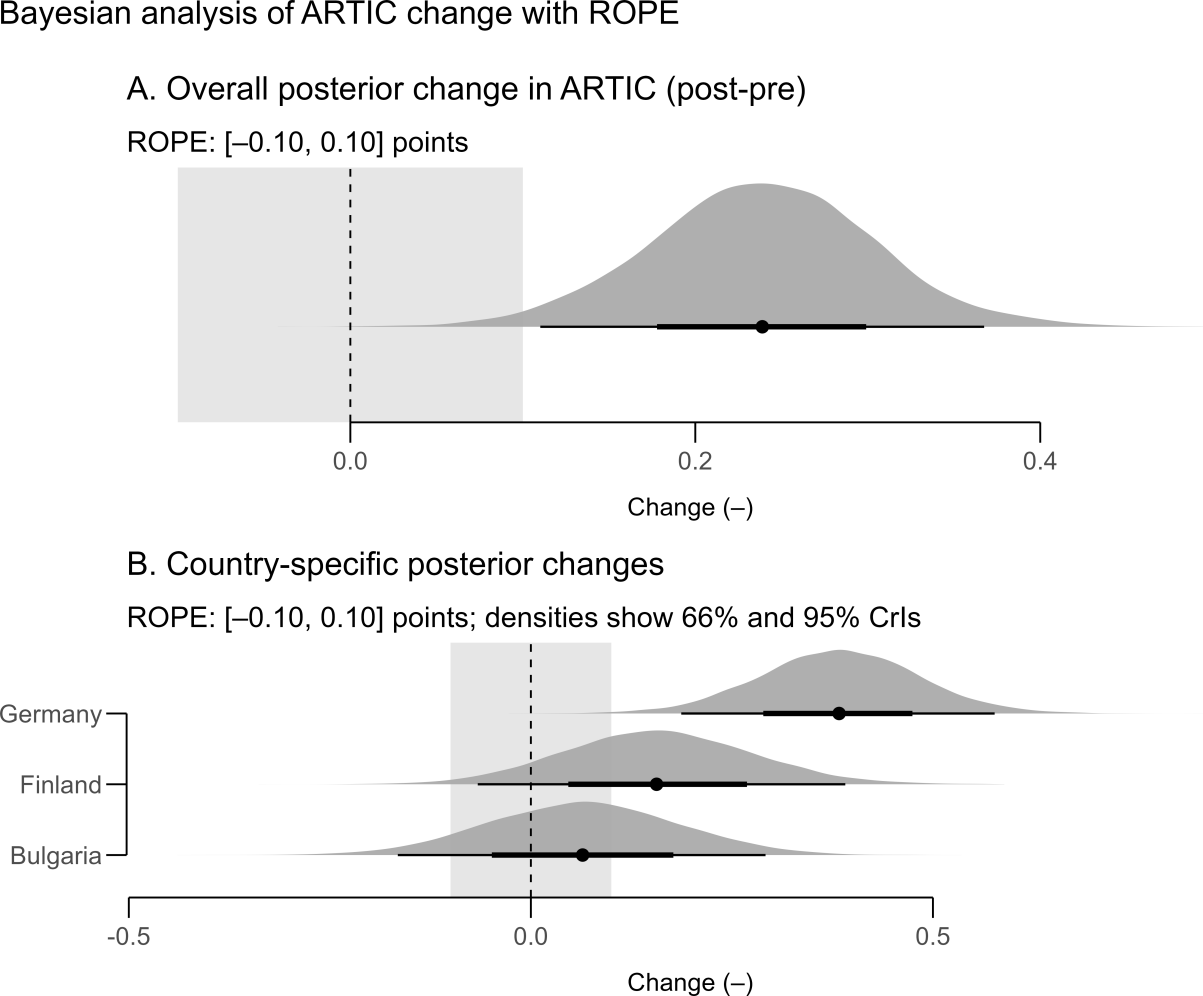
Posterior distributions of ARTIC-10 (10-item version of the Attitudes Related to Trauma-Informed Care) change (*Δ*=post-pre) with the region of practical equivalence (ROPE), overall and by country. CrI: credible interval.

A Bayesian analysis was undertaken to quantify the pre-post change in ARTIC-10 while incorporating a skeptical prior centered at zero (Figure 3). The paired change was defined for each participant as $\Delta ={\text{ARTIC}}_{post}-{\text{ARTIC}}_{pre}$. Under a normal (0, 0.3) prior on the mean change and a robust Student 2-tailed *t* test likelihood, the posterior mean Δ was estimated at 0.24 points on the 1‐7 scale, with a 95% CrI from 0.11 to 0.37. The posterior probability that the true mean change exceeded zero was effectively 1.00, whereas only 1.8% of posterior mass lay within a region of practical equivalence of ±0.10 points. The probability that Δ exceeded a practically relevant threshold of 0.20 points was 0.73.

Heterogeneity by site was examined by adding country and prior training as additive predictors of Δ. Posterior country-specific means were 0.06 points for Bulgaria (95% CrI −0.17 to 0.29; *P*[*Δ*>0]=0.71; 54% within ±0.10), 0.16 points for Finland (95% CrI −0.07 to 0.38; *P*[*Δ*>0]=0.91; 30% within ±0.10), and 0.38 points for Germany (95% CrI 0.19 to 0.58; *P*[*Δ*>0]≈1.00; 0.3% within ±0.10). These posteriors support a clear positive change in Germany, a likely but smaller positive change in Finland, and an uncertain change centered near zero in Bulgaria, mirroring the pattern observed in the frequentist analyses.

Prior trauma-related training was associated with an additional mean increase of 0.09 points (95% CrI −0.20 to 0.39), with a posterior probability of 0.73 that the training effect exceeded zero, suggesting probable, though uncertain, amplification among previously trained participants.

## Discussion

### Principal Findings

In our study, an improvement in attitudes related to TIC, as measured by the ARTIC-10 scale, was observed among students and professionals in mental health and child-welfare settings following a VR-enhanced TIC training program. The overall mean (SD) ARTIC-10 score increased from 5.33 (1.05) to 5.57 (1.20), with statistical significance (*P*<.001) and a small-to-moderate effect size. The center delivering the intervention and prior trauma-related training emerged as predictors, with participants from Germany showing greater improvement and those with prior training exhibiting higher odds of positive change. Heterogeneity across countries was noted, with significant effects limited to the German cohort, potentially influenced by their academic background and recent field placement experiences in child welfare.

### Comparison With Other Interventions

The magnitude of attitudinal change observed in this study aligns with the broader literature on TIC training interventions. A recent scoping review encompassing 157 studies found that ARTIC-based evaluations consistently demonstrate small-to-moderate improvements following diverse training modalities, including didactic curricula, experiential workshops, web-based modules, and blended approaches [49]. Across studies using pre-post designs in health care and child welfare settings, effect sizes have typically ranged from 0.2 to 0.5 SDs, with absolute score increases of approximately 0.15 to 0.30 points on the 7-point ARTIC scale [41,42,50,51]. Our overall effect (*d*=0.22; mean change 0.24 points) falls within this range, while the German cohort’s larger effect (*d*=0.56) exceeds typical benchmarks.

What distinguishes VR-enhanced training from traditional approaches is the mode of experiential engagement rather than the magnitude of effect. Conventional TIC interventions rely on seminars, case discussions, and self-directed learning; VR adds immersive, embodied simulation that may facilitate empathy development and perspective-taking through direct experiential exposure [52,53]. The comparable effect sizes suggest VR can achieve attitudinal gains similar to more time-intensive traditional formats, potentially offering efficiency advantages. The amplifying role of prior trauma-related training observed here mirrors findings elsewhere, suggesting that foundational knowledge enhances receptivity to experiential learning regardless of delivery modality [42].

VR-based interventions were evaluated as demonstrating unique advantages over non-VR methods in enhancing TIC attitudes, primarily through immersion, empathy-building, and emotional engagement. A scoping review by Tay et al [52] of 16 studies found that over half reported improvements in knowledge and attitudes toward mental illnesses, including trauma-related conditions, with VR consistently enhancing empathy in all seven relevant studies. Compared to controls, VR often yielded superior outcomes in empathy and stigma reduction, though not always in attitudes. Thorough consideration suggests VR facilitates active, embodied learning with immediate feedback, promoting retention of attitudinal skills that are difficult to achieve via passive methods [53]. In social work education, VR simulations for substance-engaged clients have built competencies in trauma-sensitive approaches, underscoring VR’s complementary role rather than its replacement for traditional training [54].

The magnitude of improvement in ARTIC-10 scores observed in this study is broadly comparable to those reported in prior ARTIC-based interventions. For instance, structured seminars and web-based approaches by Stewart et al [42] and Loomis et al [51] achieved similarly modest but statistically significant gains, demonstrating that attitudinal change toward TIC is generally incremental. What distinguishes the present VR-enhanced approach is the efficiency with which changes were achieved, as immersive simulations created embodied learning experiences without requiring extensive seminar series or multiple module completion. These findings support VR’s role as a complementary modality, capable of delivering experiential benefits similar to traditional approaches but in potentially shorter, more engaging formats.

### Strengths and Limitations

Strengths of this study include the use of a pre-post intervention design with multivariable regression analyses to control for confounders, providing robust evidence of intervention effects. The diverse sample from multiple countries (Bulgaria, Finland, and Germany) enhanced the cross-cultural perspective, while the use of the validated ARTIC-10 scale ensured reliable outcome assessment.

However, this study has several limitations that warrant consideration when interpreting its findings. The reliance on the ARTIC-10 as the sole outcome measure introduces vulnerability to self-report bias; moreover, documented psychometric limitations of the scale, particularly regarding factor structure stability and cultural invariance [46], warrant caution when interpreting cross-national comparisons. The lack of a control group prevents disentangling VR-specific effects from novelty or facilitator-driven influences. The sample size and heterogeneous participant backgrounds reduce generalizability, especially across national contexts. Practical barriers related to VR technology, including cost, accessibility, and cybersickness, may limit scalability and create inequities in who can access such training. These issues warrant caution in extrapolating the findings beyond the study’s specific settings.

This study used a single-group pre-post design with immediate posttraining measurement, which introduces several threats to internal validity. First, testing and demand effects may have occurred: participants might respond differently at postassessment because they have just completed the training or wish to align with perceived expectations. Second, history, maturation, and regression to the mean cannot be excluded without a control group. Third, social desirability and self-report bias are inherent to attitudinal scales and may inflate apparent improvements. Fourth, novelty and facilitator effects—variation in mentors across sites and the novelty of VR technology itself—could influence responses independently of the training content.

The relatively small sample size may have constrained statistical power, particularly for detecting smaller effect sizes or differences within subgroups, such as those based on program delivery center or professional experience. While the case-crossover design mitigated interindividual variability, a larger sample could enhance the precision and robustness of findings, especially for exploring contextual or demographic influences. The small proportion of male participants (n=8, 10%) also limits the generalizability of findings across sexes.

Second, the generalizability of the results may be limited due to the study’s focus on specific countries, centers (Germany, Finland, and Bulgaria), and settings (residential child and adolescent care and child psychiatric wards). Although the VR intervention was designed to be universally applicable, cultural, organizational, or systemic differences across countries and care settings may restrict the applicability of the findings to other contexts, such as nonresidential care or regions with distinct health care infrastructures.

Third, potential biases may have influenced the outcomes. Selection bias is a concern, as convenience sampling may have resulted in a participant pool that was more motivated or receptive to VR-based training, potentially inflating observed improvements in ARTIC-10 scores. Additionally, social desirability bias may have affected self-reported ARTIC-10 scores, particularly in postintervention assessments, as participants might have provided responses aligned with trauma-informed principles due to the training context or mentor presence.

Finally, limitations related to VR technology itself must be acknowledged. Accessibility issues, such as limited availability, technological variability of VR equipment, or inadequate technical infrastructure in some settings, may hinder the scalability and equitable implementation of the intervention. Furthermore, variability in participants’ comfort with VR technology, prior exposure, or technical challenges (eg, motion sickness and interface difficulties) may have introduced variability in the intervention’s effectiveness, potentially affecting the consistency of outcomes across participants. Finally, without long-term follow-up, it remains unclear whether the gains observed represent lasting attitudinal change or short-term responses to the intervention context.

### Implications for Practice and Future Research

The critical next step for this line of research is comparative evaluation. Future trials should randomize participants to VR-enhanced versus standard (non-VR) TIC training conditions, matched for contact time and content, to isolate the specific contribution of immersive simulation. Such designs would address whether VR adds value beyond conventional approaches or simply represents an alternative delivery mode with equivalent outcomes.

Beyond comparative effectiveness, several methodological advances are warranted. First, longitudinal follow-up assessments at 3, 6, and 12 months postintervention would establish whether attitudinal gains are durable or decay over time. Second, behavioral outcome measures—such as direct observation of staff-child interactions, documented use of de-escalation techniques, or rates of restrictive practice use—would determine whether attitudinal improvements translate into practice change. Third, nested qualitative components (eg, semistructured interviews and focus groups) could illuminate mechanisms of effect and identify barriers to skill transfer. Fourth, process evaluation embedded within future trials should capture implementation fidelity, participant engagement metrics, and contextual factors that may moderate effectiveness.

The observed site-level heterogeneity underscores the need for studies that are adequately powered to examine moderators of intervention response, including baseline TIC exposure, professional background, and organizational readiness. Multilevel designs that account for clustering within sites and mentors would strengthen causal inference. Finally, cost-effectiveness analyses comparing VR-enhanced training to conventional approaches would inform resource allocation decisions for workforce development in child welfare and mental health settings.

### Conclusions

This multisite evaluation provides preliminary evidence that a VR-enhanced TIC training program can improve attitudes toward TIC among emerging and practicing professionals in child and adolescent residential and mental health settings. The relatively small, predominantly female sample and the potential for selection and self-report bias warrant caution in interpretation, and the heterogeneity of results across countries highlights the need for approaches tailored to cultural, professional, and systemic contexts.

Nevertheless, the significant improvement observed in Germany (*d*=0.56) and the amplifying effect of prior trauma-related training suggest that VR-enhanced approaches may be most effective when integrated into scaffolded curricula that build upon learners’ existing knowledge. These findings support the role of immersive, experiential methods as a complementary tool within broader TIC education frameworks. Comparative trials with non-VR controls are now needed to determine whether VR confers specific advantages and to guide evidence-based workforce development in TIC.

## Supplementary material

10.2196/86543 Multimedia Appendix 1 Safe4Child Intervention: curriculum, VR simulation, and delivery details.

10.2196/86543 Multimedia Appendix 2 Reproducible code with simulated data.

10.2196/86543 Checklist 1 TREND Checklist.

10.2196/86543 Checklist 2 TiDieR Checklist.

## Abbreviations

ARTIC-10: 10-item version of the Attitudes Related to Trauma-Informed Care
GDPR: General Data Protection Regulation
HAW: Hamburg University of Applied Sciences
MUP: Medical University of Plovdiv
ROPE: region of practical equivalence
TIC: trauma-informed care
TIDieR: Template for Intervention Description and Replication
TREND: Transparent Reporting of Evaluations with Non-Randomized Designs
TUAS: Turku University of Applied Sciences
VR: virtual reality
