# Supplementary material for: Virtual Reality–Enhanced Training for Trauma-Informed Care Among Residential and Child Mental Health Professionals: Pre-Post Evaluation Study
Source: JMIR Med Educ. 2026 Apr 17;12:e86543. doi: 10.2196/86543 (PMC13089627; doi:10.2196/86543)

**Supplement 1. Safe4Child Intervention: Curriculum, VR Simulation, and Delivery Details**

**Figure S1.** Immersive VR training scenario (residential care): initial scene showing an approximately 8-year-old boy seated and disengaged after a missed caregiver visit. The screenshot captures the trainee’s first contact; later segments depict escalating distress, including episodes of self-directed and outward aggression toward the trainee.


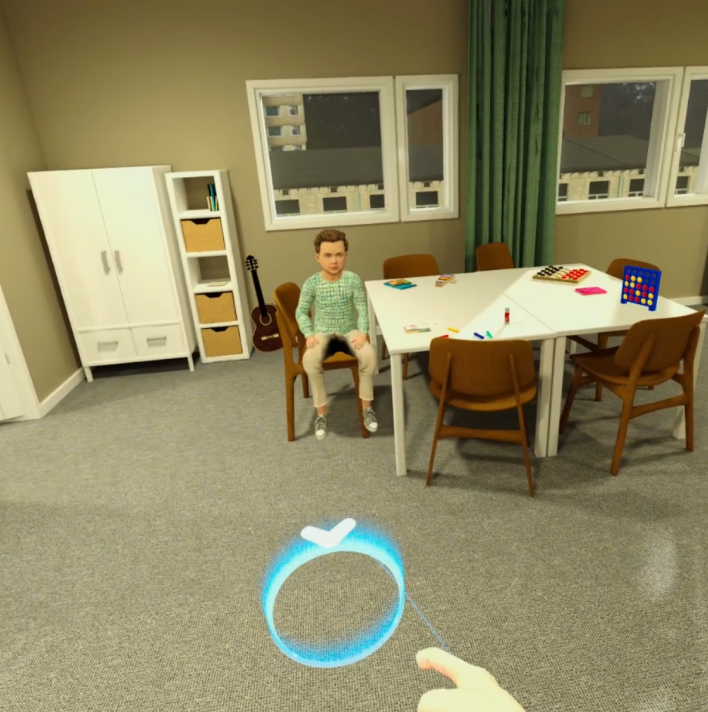


**Figure S2.** Online learning platform (Moodle) course interface for the TIC curriculum. The screenshot shows the left-hand navigation column listing sequenced modules and embedded quizzes for reinforced learning and self-checks, while the right-hand content pane displays an example lesson with interactive materials—readings, embedded videos, and slide presentations—presented in a scrollable viewer. Within the lesson, learners encounter inline prompts for reflection and links to supplementary resources, with progress indicators and clear next-step cues to support stepwise completion. This layout was used to standardize the pre-VR preparation across sites, ensuring that participants engaged with common core content before undertaking the immersive simulation.


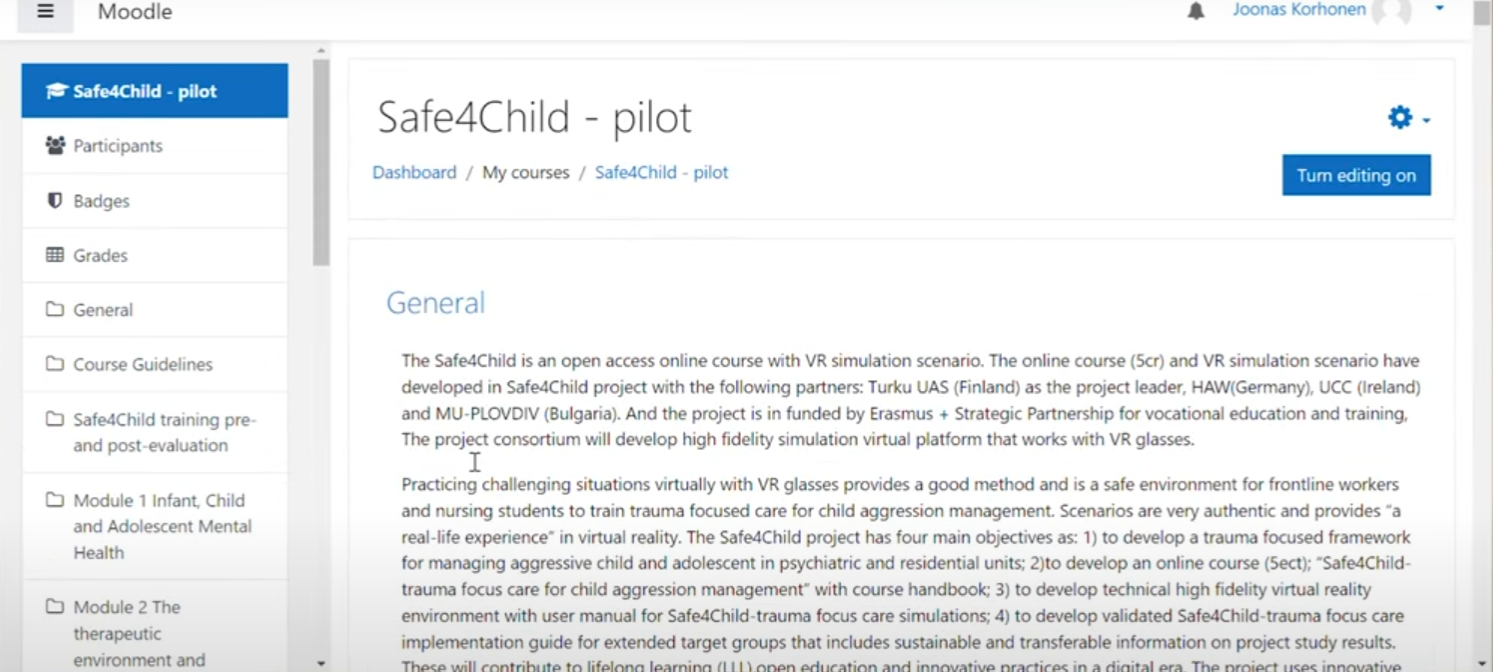


**Figure S3.** VR simulation setup and facilitation. A volunteer researcher demonstrates the standard study configuration: the participant stands within a clear ~1-meter radius while wearing a VR headset and holding two handheld controllers used for navigation and object interaction. The left side of the figure shows the participant’s live field of view mirrored to a laptop/monitor, allowing the facilitator (positioned at the workstation in the foreground) to supervise the session, provide standardized prompts, trigger scene transitions, and pause or end the simulation if needed for comfort or safety. The open floor space minimizes collisions and allows natural arm movements during interaction; the controller layout supports pointing, selection, and basic locomotion consistent with the training scenario. This arrangement—participant, mirrored display, and facilitator oversight—was applied uniformly across study sites to ensure a consistent training environment and comparable user experience. The individual depicted is a volunteer; informed consent was obtained for participation, photography/screen capture, and publication, and identifying details have been minimized.


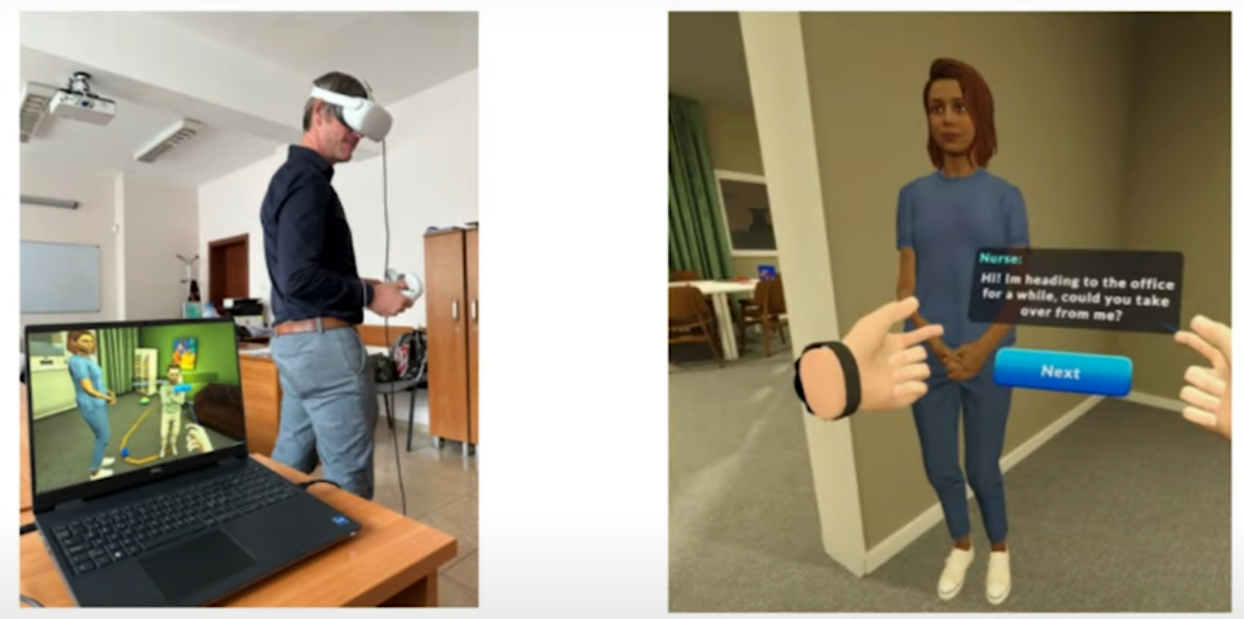

Supplement: Multimedia Appendix 1 [file mededu-v12-e86543-s001.docx]
