# Supplementary material for: Virtual Reality–Enhanced Training for Trauma-Informed Care Among Residential and Child Mental Health Professionals: Pre-Post Evaluation Study
Source: JMIR Med Educ. 2026 Apr 17;12:e86543. doi: 10.2196/86543 (PMC13089627; doi:10.2196/86543)
Supplement: Multimedia Appendix 2 [file mededu-v12-e86543-s002.docx]

**Supplement 2**

**Reproducible Code with Simulated Data**

# 1. Overview

This document programmatically generates a **synthetic dataset that mimics the structure and signal of the study data** (same variables and similar effect sizes), then reproduces the full analysis pipeline: descriptive summaries, paired pre–post tests overall and by country, proportion improved, linear and logistic regressions, Bayesian estimation with ROPE, and internal consistency (Cronbach’s alpha) overall and by country. **No real participant data are used.**

# 2. Data generation

# --- Reproducible data generation for Supplement 2 ---
set.seed(2025)

# countries and sample sizes (sum to 79)
n_by_country <- c(Bulgaria = 40, Finland = 20, Germany = 19)
countries <- rep(names(n_by_country), n_by_country)

# ids like "BG06", "FI21", "D71"
code_map <- c(Bulgaria = "BG", Finland = "FI", Germany = "D")
id <- unlist(Map(function(cc, n){
 paste0(code_map[cc], sprintf("%02d", seq_len(n)))
}, names(n_by_country), n_by_country))

# demographics and covariates
male <- rbinom(length(countries), 1, prob = 0.18)
age <- round(pmin(pmax(rnorm(length(countries), 27, 7), 19), 60))
training <- rbinom(length(countries), 1, prob = 0.30)

helthcare_expiriance_yrs <- pmax(0, round(rlnorm(length(countries), log(1.5), 0.8), 1))
children_exp_yrs <- pmax(0, round(rlnorm(length(countries), log(1.0), 0.9), 1))

education_levels <- c("Student","Diploma","Bachelor's Degree","Master's Degree","Doctorate")
education <- sample(education_levels, length(countries), replace = TRUE,
 prob = c(.55,.10,.15,.15,.05))

org_levels <- c("None","Mental health","Paediatric","Child-welfare (residential)")
type_organisation <- sample(org_levels, length(countries), replace = TRUE,
 prob = c(.55,.20,.10,.15))

# --- Latent pre/post attitude ---
mu_pre_country <- c(Bulgaria = 5.1, Finland = 5.4, Germany = 5.3)
mu_delta_country <- c(Bulgaria = 0.06, Finland = 0.16, Germany = 0.38)
delta_training <- 0.09

latent_pre <- rnorm(length(countries), mu_pre_country[countries], 0.5)
latent_post <- latent_pre +
 rnorm(length(countries), mu_delta_country[countries], 0.25) +
 delta_training * training

# --- Item model and reverse scoring ---
J <- 10
rev_idx <- c(2,4,6,8,9)
a_j <- rnorm(J, 0, 0.15)
disc_1_7 <- function(x) pmin(7, pmax(1, round(x)))

make_items <- function(latent, suffix){
 N <- length(latent)
 M <- outer(latent, a_j, "+") + matrix(rnorm(N * J, 0, 0.5), nrow = N, ncol = J)
 M <- matrix(disc_1_7(M), nrow = N, ncol = J,
 dimnames = list(NULL, paste0("ARTIC_item", 1:J, suffix)))
 M
}

items_pre_mat <- make_items(latent_pre, "pre")
items_post_mat <- make_items(latent_post, "post")

# reverse by name (robust)
items_pre_mat[, paste0("ARTIC_item", rev_idx, "pre")] <- 8 - items_pre_mat[, paste0("ARTIC_item", rev_idx, "pre")]
items_post_mat[, paste0("ARTIC_item", rev_idx, "post")] <- 8 - items_post_mat[, paste0("ARTIC_item", rev_idx, "post")]

# --- Assemble analysis frames ---
base_df <- tibble::tibble(
 id, country = countries, male, age, education, helthcare_expiriance_yrs,
 children_exp_yrs, training, type_organisation
)

d_wide <- dplyr::bind_cols(
 base_df,
 tibble::as_tibble(items_pre_mat),
 tibble::as_tibble(items_post_mat)
) |>
 dplyr::rowwise() |>
 dplyr::mutate(
 artic_pre = mean(dplyr::c_across(tidyselect::ends_with("pre")), na.rm = TRUE),
 artic_post = mean(dplyr::c_across(tidyselect::ends_with("post")), na.rm = TRUE),
 increase = artic_post - artic_pre,
 increase_fct = ifelse(increase > 0, "increase", "decrease")
 ) |>
 dplyr::ungroup()

d_artic <- d_wide |>
 dplyr::select(id, country, male, age, education, helthcare_expiriance_yrs,
 children_exp_yrs, training, type_organisation,
 dplyr::starts_with("ARTIC_item"),
 artic_pre, artic_post, increase, increase_fct) |>
 tidyr::pivot_longer(
 cols = c(artic_pre, artic_post),
 names_to = "condition", values_to = "score"
 ) |>
 dplyr::mutate(condition = ifelse(condition == "artic_pre", "artic_pre", "artic_post"))

# 3. Descriptive statistics

d_wide |>
 select(country, male, age, education, helthcare_expiriance_yrs,
 children_exp_yrs, training, type_organisation) |>
 mutate(
 male = factor(male, levels = c(0,1), labels = c("Female","Male")),
 training = factor(training, levels = c(0,1), labels = c("No","Yes"))
 ) |>
 tbl_summary(
 by = country,
 missing = "no",
 statistic = list(
 all_continuous() ~ "{mean} ({sd})",
 all_categorical() ~ "{n} / {N} ({p}%)"
 ),
 digits = list(all_continuous() ~ 1)
 ) |>
 add_overall() |>
 add_p()

| **Characteristic** | **Overall**, N = 79^1^ | **Bulgaria**, N = 40^1^ | **Finland**, N = 20^1^ | **Germany**, N = 19^1^ | **p-value**^2^ |
| --- | --- | --- | --- | --- | --- |
| male |  |  |  |  | 0.8 |
| Female | 67 / 79 (85%) | 33 / 40 (83%) | 18 / 20 (90%) | 16 / 19 (84%) |  |
| Male | 12 / 79 (15%) | 7 / 40 (18%) | 2 / 20 (10%) | 3 / 19 (16%) |  |
| age | 28.8 (6.7) | 29.0 (6.2) | 29.0 (6.6) | 28.1 (8.0) | 0.8 |
| education |  |  |  |  | 0.3 |
| Bachelor's Degree | 10 / 79 (13%) | 5 / 40 (13%) | 4 / 20 (20%) | 1 / 19 (5.3%) |  |
| Diploma | 8 / 79 (10%) | 7 / 40 (18%) | 0 / 20 (0%) | 1 / 19 (5.3%) |  |
| Doctorate | 9 / 79 (11%) | 5 / 40 (13%) | 3 / 20 (15%) | 1 / 19 (5.3%) |  |
| Master's Degree | 13 / 79 (16%) | 4 / 40 (10%) | 4 / 20 (20%) | 5 / 19 (26%) |  |
| Student | 39 / 79 (49%) | 19 / 40 (48%) | 9 / 20 (45%) | 11 / 19 (58%) |  |
| helthcare_expiriance_yrs | 2.1 (1.5) | 2.5 (1.8) | 1.9 (1.3) | 1.4 (0.7) | 0.029 |
| children_exp_yrs | 1.5 (1.5) | 1.8 (1.9) | 1.3 (1.0) | 1.2 (1.1) | 0.4 |
| training | 19 / 79 (24%) | 6 / 40 (15%) | 9 / 20 (45%) | 4 / 19 (21%) | 0.046 |
| type_organisation |  |  |  |  | 0.8 |
| Child-welfare (residential) | 9 / 79 (11%) | 3 / 40 (7.5%) | 3 / 20 (15%) | 3 / 19 (16%) |  |
| Mental health | 14 / 79 (18%) | 7 / 40 (18%) | 4 / 20 (20%) | 3 / 19 (16%) |  |
| None | 46 / 79 (58%) | 23 / 40 (58%) | 12 / 20 (60%) | 11 / 19 (58%) |  |
| Paediatric | 10 / 79 (13%) | 7 / 40 (18%) | 1 / 20 (5.0%) | 2 / 19 (11%) |  |
| ^1^n / N (%); Mean (SD) | | | | | |
| ^2^Fisher's exact test; Kruskal-Wallis rank sum test | | | | | |

# 4. Frequentist pre–post comparisons

# Overall paired tests (wide -> base tests)
overall_t <- broom::tidy(
 t.test(d_wide$artic_post, d_wide$artic_pre, paired = TRUE)
)
overall_w <- broom::tidy(
 wilcox.test(d_wide$artic_post, d_wide$artic_pre, paired = TRUE, exact = FALSE)
)

overall_t

# A tibble: 1 × 8
 estimate statistic p.value parameter conf.low conf.high method alternative
 <dbl> <dbl> <dbl> <dbl> <dbl> <dbl> <chr> <chr>
1 -0.0418 -1.55 0.125 78 -0.0954 0.0119 Paired t-… two.sided

overall_w

# A tibble: 1 × 4
 statistic p.value method alternative
 <dbl> <dbl> <chr> <chr>
1 790. 0.135 Wilcoxon signed rank test with continuity corre… two.sided

# By-country paired t-tests
t_by_cty <- d_wide |>
 dplyr::group_by(country) |>
 dplyr::summarise(
 res = list(broom::tidy(t.test(artic_post, artic_pre, paired = TRUE))),
 .groups = "drop"
 ) |>
 tidyr::unnest(res)

t_by_cty

# A tibble: 3 × 9
 country estimate statistic p.value parameter conf.low conf.high method
 <chr> <dbl> <dbl> <dbl> <dbl> <dbl> <dbl> <chr>
1 Bulgaria -0.0375 -0.896 0.376 39 -0.122 0.0472 Paired t-test
2 Finland -0.0400 -0.867 0.397 19 -0.137 0.0565 Paired t-test
3 Germany -0.0526 -1.01 0.326 18 -0.162 0.0568 Paired t-test
# ℹ 1 more variable: alternative <chr>

# By-country paired Wilcoxon tests
w_by_cty <- d_wide |>
 dplyr::group_by(country) |>
 dplyr::summarise(
 res = list(broom::tidy(wilcox.test(artic_post, artic_pre, paired = TRUE, exact = FALSE))),
 .groups = "drop"
 ) |>
 tidyr::unnest(res)

w_by_cty

# A tibble: 3 × 5
 country statistic p.value method alternative
 <chr> <dbl> <dbl> <chr> <chr>
1 Bulgaria 262 0.389 Wilcoxon signed rank test with continu… two.sided
2 Finland 51 0.392 Wilcoxon signed rank test with continu… two.sided
3 Germany 24.5 0.267 Wilcoxon signed rank test with continu… two.sided

# Proportion with increased score by country + tests
tab_inc <- d_wide |>
 dplyr::mutate(inc_bin = increase > 0) |>
 dplyr::count(country, inc_bin) |>
 tidyr::pivot_wider(names_from = inc_bin, values_from = n, values_fill = 0) |>
 dplyr::rename(decrease = `FALSE`, increase = `TRUE`) |>
 dplyr::mutate(total = increase + decrease)

tab_inc

# A tibble: 3 × 4
 country decrease increase total
 <chr> <int> <int> <int>
1 Bulgaria 24 16 40
2 Finland 14 6 20
3 Germany 15 4 19

# Chi-square test of independence (2×k table)
chisq_res <- chisq.test(as.matrix(tab_inc[, c("increase","decrease")]))
chisq_res

Pearson's Chi-squared test

data: as.matrix(tab_inc[, c("increase", "decrease")])
X-squared = 2.1972, df = 2, p-value = 0.3333

# Alternatively: k-sample test for equal proportions
prop_res <- prop.test(x = tab_inc$increase, n = tab_inc$total)
prop_res

3-sample test for equality of proportions without continuity correction

data: tab_inc$increase out of tab_inc$total
X-squared = 2.1972, df = 2, p-value = 0.3333
alternative hypothesis: two.sided
sample estimates:
 prop 1 prop 2 prop 3
0.4000000 0.3000000 0.2105263

# 5. Plots: distribution of change and proportion improved

# Build long table for plotting + proportions
inc_long <- d_wide |>
 dplyr::mutate(
 increase_fct = factor(ifelse(increase > 0, "increase", "decrease"),
 levels = c("decrease","increase"))
 ) |>
 dplyr::count(country, increase_fct, .drop = FALSE) |>
 dplyr::group_by(country) |>
 dplyr::mutate(total = sum(n), prop = n / total) |>
 dplyr::ungroup()

# Data for the bar plot (just the "increase" rows)
prop_country <- dplyr::filter(inc_long, increase_fct == "increase")

# Wide table for tests
inc_wide <- inc_long |>
 dplyr::select(country, increase_fct, n) |>
 tidyr::pivot_wider(names_from = increase_fct, values_from = n, values_fill = 0) |>
 dplyr::mutate(total = increase + decrease)

# Tests
chisq_res <- chisq.test(as.matrix(inc_wide[, c("increase","decrease")]))
prop_res <- prop.test(x = inc_wide$increase, n = inc_wide$total)

chisq_res

Pearson's Chi-squared test

data: as.matrix(inc_wide[, c("increase", "decrease")])
X-squared = 2.1972, df = 2, p-value = 0.3333

prop_res

3-sample test for equality of proportions without continuity correction

data: inc_wide$increase out of inc_wide$total
X-squared = 2.1972, df = 2, p-value = 0.3333
alternative hypothesis: two.sided
sample estimates:
 prop 1 prop 2 prop 3
0.4000000 0.3000000 0.2105263

# Plot
p2 <- ggplot(prop_country,
 aes(y = forcats::fct_reorder(country, prop),
 x = prop, fill = country)) +
 geom_col(alpha = .75, color = "black") +
 geom_text(aes(label = paste0(n, " (", scales::percent(prop, accuracy = 0.1), ")")),
 hjust = -0.05) +
 scale_x_continuous(labels = scales::percent, limits = c(0, 1)) +
 labs(x = NULL, y = NULL,
 subtitle = "Proportion of participants with increased ARTIC score") +
 theme_minimal() + theme(legend.position = "none")

p2


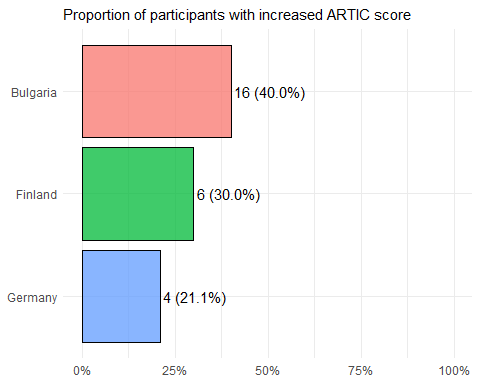


# 6. Regression models

# Linear regression: continuous change
fit_lm <- lm(increase ~ country + male + age + (helthcare_expiriance_yrs > 0) + training, data = d_wide)
broom::tidy(fit_lm, conf.int = TRUE)

# A tibble: 7 × 7
 term estimate std.error statistic p.value conf.low conf.high
 <chr> <dbl> <dbl> <dbl> <dbl> <dbl> <dbl>
1 (Intercept) -4.10e-2 0.129 -0.318 0.751 -0.298 0.216
2 countryFinland -1.52e-2 0.0714 -0.213 0.832 -0.157 0.127
3 countryGermany -1.77e-2 0.0690 -0.256 0.799 -0.155 0.120
4 male -2.82e-2 0.0795 -0.355 0.724 -0.187 0.130
5 age 1.09e-4 0.00420 0.0260 0.979 -0.00825 0.00847
6 helthcare_expiriance_… NA NA NA NA NA NA
7 training 3.52e-2 0.0696 0.506 0.614 -0.103 0.174

# Logistic regression: increased vs. not
d_wide$inc_bin <- as.integer(d_wide$increase > 0)
fit_glm <- glm(inc_bin ~ country + male + age + (helthcare_expiriance_yrs > 0) + training,
 data = d_wide, family = binomial())
b_tidy <- broom::tidy(fit_glm, conf.int = TRUE, exponentiate = TRUE)
b_tidy

# A tibble: 7 × 7
 term estimate std.error statistic p.value conf.low conf.high
 <chr> <dbl> <dbl> <dbl> <dbl> <dbl> <dbl>
1 (Intercept) 1.23 1.14 0.178 0.859 0.130 12.0
2 countryFinland 0.533 0.627 -1.00 0.316 0.146 1.76
3 countryGermany 0.371 0.660 -1.50 0.133 0.0904 1.27
4 male 0.539 0.747 -0.828 0.408 0.105 2.14
5 age 0.980 0.0377 -0.532 0.595 0.908 1.05
6 helthcare_expiriance_… NA NA NA NA NA NA
7 training 1.60 0.608 0.774 0.439 0.476 5.34

# Clean + reorder terms for plotting
library(forcats)
library(stringr)

## Linear model
tidy_lm <- broom::tidy(fit_lm, conf.int = TRUE) |>
 dplyr::filter(term != "(Intercept)") |>
 dplyr::mutate(
 term_label = dplyr::case_when(
 term == "countryFinland" ~ "Finland vs Bulgaria",
 term == "countryGermany" ~ "Germany vs Bulgaria",
 term == "male" ~ "Male vs Female",
 str_detect(term, "helthcare_expiriance") ~ "Any healthcare experience",
 term == "training" ~ "Prior trauma-related training",
 TRUE ~ term
 )
 ) |>
 dplyr::mutate(term_f = forcats::fct_reorder(factor(term_label), estimate))

plt_lm <- ggplot(tidy_lm, aes(x = estimate, y = term_f)) +
 geom_point() +
 geom_errorbarh(aes(xmin = conf.low, xmax = conf.high), height = 0.2) +
 geom_vline(xintercept = 0, linetype = 2) +
 labs(x = "Estimate (95% CI)", y = NULL, subtitle = "Linear model: ARTIC change") +
 theme_minimal()

## Logistic model (ORs)
tidy_glm <- broom::tidy(fit_glm, conf.int = TRUE, exponentiate = TRUE) |>
 dplyr::filter(term != "(Intercept)") |>
 dplyr::mutate(
 term_label = dplyr::case_when(
 term == "countryFinland" ~ "Finland vs Bulgaria",
 term == "countryGermany" ~ "Germany vs Bulgaria",
 term == "male" ~ "Male vs Female",
 str_detect(term, "helthcare_expiriance") ~ "Any healthcare experience",
 term == "training" ~ "Prior trauma-related training",
 TRUE ~ term
 )
 ) |>
 dplyr::mutate(term_f = forcats::fct_reorder(factor(term_label), estimate))

plt_glm <- ggplot(tidy_glm, aes(x = estimate, y = term_f)) +
 geom_point() +
 geom_errorbarh(aes(xmin = conf.low, xmax = conf.high), height = 0.2) +
 geom_vline(xintercept = 1, linetype = 2) +
 labs(x = "Odds ratio (95% CI)", y = NULL, subtitle = "Logistic model: probability of increase") +
 theme_minimal()

plt_lm + plt_glm


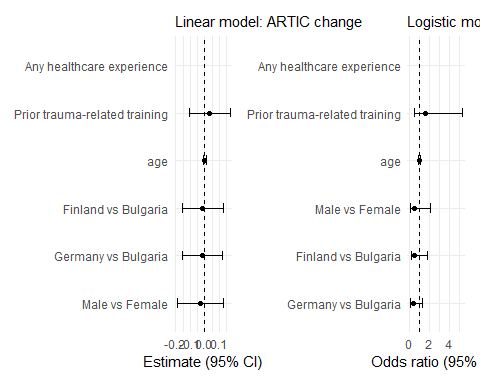


# 7. Bayesian estimation with ROPE

We quantify the paired change $\Delta=\text{ARTIC}_{post}-\text{ARTIC}_{pre}$ under weakly skeptical priors centered at zero. The region of practical equivalence (ROPE) encodes a range of changes considered too small to matter on the 1–7 scale; here we use $\pm0.10$ points.

# ROPE
rope_low <- -0.10
rope_high <- 0.10

# Overall model: Δ_i ~ Student-t(μ, σ, ν), μ = α
pri_overall <- c(
 prior(normal(0, 0.3), class = Intercept),
 prior(student_t(3, 0, 0.7), class = sigma)
)

delta_df <- d_wide |>
 transmute(id, country, training, delta = artic_post - artic_pre)

fit_delta <- brm(
 delta ~ 1,
 data = delta_df,
 family = student(),
 prior = pri_overall,
 chains = 4, iter = 4000, warmup = 1000,
 control = list(adapt_delta = 0.98, max_treedepth = 12),
 refresh = 0, seed = 2025
)

summary(fit_delta)

Family: student
 Links: mu = identity; sigma = identity; nu = identity
Formula: delta ~ 1
 Data: delta_df (Number of observations: 79)
 Draws: 4 chains, each with iter = 4000; warmup = 1000; thin = 1;
 total post-warmup draws = 12000

Regression Coefficients:
 Estimate Est.Error l-95% CI u-95% CI Rhat Bulk_ESS Tail_ESS
Intercept -0.04 0.03 -0.10 0.01 1.00 7347 6482

Further Distributional Parameters:
 Estimate Est.Error l-95% CI u-95% CI Rhat Bulk_ESS Tail_ESS
sigma 0.23 0.02 0.19 0.28 1.00 6705 6763
nu 23.63 14.32 5.89 60.43 1.00 6588 5936

Draws were sampled using sampling(NUTS). For each parameter, Bulk_ESS
and Tail_ESS are effective sample size measures, and Rhat is the potential
scale reduction factor on split chains (at convergence, Rhat = 1).

# Posterior summaries and probabilities
post <- as_draws_df(fit_delta)
post_summ <- post |>
 summarise(
 mean = mean(b_Intercept),
 l95 = quantile(b_Intercept, .025),
 u95 = quantile(b_Intercept, .975),
 p_gt0 = mean(b_Intercept > 0),
 p_in_rope = mean(b_Intercept > rope_low & b_Intercept < rope_high),
 p_gt0p2 = mean(b_Intercept > 0.20)
 )
post_summ

# A tibble: 1 × 6
 mean l95 u95 p_gt0 p_in_rope p_gt0p2
 <dbl> <dbl> <dbl> <dbl> <dbl> <dbl>
1 -0.0427 -0.0968 0.0110 0.0598 0.981 0

# Moderated model: country + training
delta_df2 <- delta_df |>
 mutate(
 country = factor(country, levels = c("Bulgaria","Finland","Germany")),
 training = factor(training, levels = c(0,1), labels = c("No","Yes"))
 )

pri_mod <- c(
 prior(normal(0, 0.3), class = Intercept),
 prior(normal(0, 0.3), class = b),
 prior(student_t(3, 0, 0.7), class = sigma)
)

fit_delta_mod <- brm(
 delta ~ 1 + country + training,
 data = delta_df2,
 family = student(),
 prior = pri_mod,
 chains = 4, iter = 4000, warmup = 1000,
 control = list(adapt_delta = 0.99, max_treedepth = 12),
 refresh = 0, seed = 2025
)

summary(fit_delta_mod)

Family: student
 Links: mu = identity; sigma = identity; nu = identity
Formula: delta ~ 1 + country + training
 Data: delta_df2 (Number of observations: 79)
 Draws: 4 chains, each with iter = 4000; warmup = 1000; thin = 1;
 total post-warmup draws = 12000

Regression Coefficients:
 Estimate Est.Error l-95% CI u-95% CI Rhat Bulk_ESS Tail_ESS
Intercept -0.04 0.04 -0.12 0.03 1.00 12201 7909
countryFinland -0.01 0.07 -0.15 0.12 1.00 11922 8930
countryGermany -0.01 0.07 -0.14 0.12 1.00 12561 8866
trainingYes 0.03 0.07 -0.10 0.16 1.00 12061 8490

Further Distributional Parameters:
 Estimate Est.Error l-95% CI u-95% CI Rhat Bulk_ESS Tail_ESS
sigma 0.23 0.02 0.19 0.28 1.00 10239 7534
nu 23.33 14.16 5.81 59.03 1.00 10531 7547

Draws were sampled using sampling(NUTS). For each parameter, Bulk_ESS
and Tail_ESS are effective sample size measures, and Rhat is the potential
scale reduction factor on split chains (at convergence, Rhat = 1).

# Country-specific posterior draws for Δ
site_df <- fit_delta_mod |>
 tidybayes::spread_draws(b_Intercept, b_countryFinland, b_countryGermany) |>
 mutate(
 Bulgaria = b_Intercept,
 Finland = b_Intercept + b_countryFinland,
 Germany = b_Intercept + b_countryGermany
 ) |>
 select(Bulgaria, Finland, Germany) |>
 pivot_longer(everything(), names_to = "site", values_to = "delta") |>
 mutate(site = factor(site, levels = c("Bulgaria","Finland","Germany")))

# Summaries (for table if needed)
site_summ <- site_df |>
 group_by(site) |>
 summarise(
 mean = mean(delta),
 l95 = quantile(delta, .025),
 u95 = quantile(delta, .975),
 p_gt0 = mean(delta > 0),
 p_inRP = mean(delta > rope_low & delta < rope_high),
 .groups = "drop"
)
site_summ

# A tibble: 3 × 6
 site mean l95 u95 p_gt0 p_inRP
 <fct> <dbl> <dbl> <dbl> <dbl> <dbl>
1 Bulgaria -0.0438 -0.122 0.0347 0.129 0.922
2 Finland -0.0560 -0.174 0.0596 0.173 0.765
3 Germany -0.0566 -0.168 0.0550 0.154 0.777

# ROPE plots: overall and by country
overall_df <- as_draws_df(fit_delta) |>
 transmute(delta = b_Intercept, group = "Overall")

p_overall <- ggplot(overall_df, aes(x = delta, y = group)) +
 annotate("rect", xmin = rope_low, xmax = rope_high, ymin = -Inf, ymax = Inf, alpha = 0.15) +
 ggdist::stat_halfeye(.width = c(0.66, 0.95), slab_alpha = 0.9) +
 geom_vline(xintercept = 0, linetype = 2) +
 labs(title = "Overall posterior change in ARTIC (post − pre)",
 subtitle = sprintf("ROPE: [%.2f, %.2f] points", rope_low, rope_high),
 x = "Change (Δ)", y = NULL) +
 theme_minimal() +
 theme(axis.text.y = element_blank(), axis.ticks.y = element_blank())

p_countries <- ggplot(site_df, aes(x = delta, y = site)) +
 annotate("rect", xmin = rope_low, xmax = rope_high, ymin = -Inf, ymax = Inf, alpha = 0.15) +
 ggdist::stat_halfeye(.width = c(0.66, 0.95), slab_alpha = 0.9) +
 geom_vline(xintercept = 0, linetype = 2) +
 labs(title = "Country-specific posterior changes",
 subtitle = sprintf("ROPE: [%.2f, %.2f]; densities show 66%% and 95%% CrIs", rope_low, rope_high),
 x = "Change (Δ)", y = NULL) +
 theme_minimal()

p_bayes <- p_overall / p_countries + patchwork::plot_annotation(
 title = "Bayesian analysis of ARTIC change with ROPE"
)
p_bayes


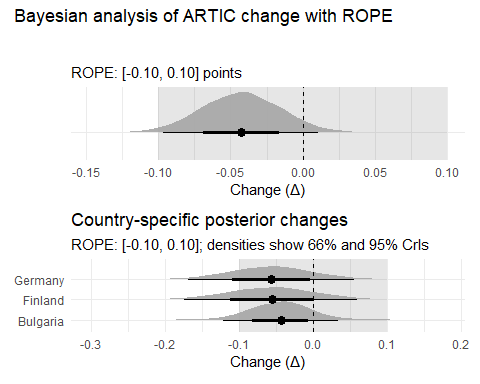


# 8. Reliability: Cronbach’s alpha (ARTIC-10)

# item names
items_pre <- paste0("ARTIC_item", 1:10, "pre")
items_post <- paste0("ARTIC_item", 1:10, "post")

alpha_wrap <- function(df_items){
 fit <- psych::alpha(df_items, check.keys = FALSE, warnings = FALSE, use = "pairwise")
 tibble(
 alpha = unname(fit$total$std.alpha),
 se = unname(fit$total$ase),
 l95 = pmax(0, alpha - 1.96*se),
 u95 = pmin(1, alpha + 1.96*se),
 k = ncol(df_items)
 )
}

# overall
alpha_overall_pre <- alpha_wrap(select(d_wide, all_of(items_pre))) |> mutate(country = "Overall", time = "Pre")
alpha_overall_post <- alpha_wrap(select(d_wide, all_of(items_post))) |> mutate(country = "Overall", time = "Post")

# by-country
alpha_by_country_pre <- d_wide |>
 group_by(country) |>
 group_modify(~ alpha_wrap(select(.x, all_of(items_pre)))) |>
 mutate(time = "Pre") |>
 ungroup()

alpha_by_country_post <- d_wide |>
 group_by(country) |>
 group_modify(~ alpha_wrap(select(.x, all_of(items_post)))) |>
 mutate(time = "Post") |>
 ungroup()

alpha_results <- bind_rows(
 alpha_overall_pre,
 alpha_overall_post,
 alpha_by_country_pre,
 alpha_by_country_post
) |>
 select(alpha, se, l95, u95, k, country, time)

alpha_results

# A tibble: 8 × 7
 alpha se l95 u95 k country time
 <dbl> <dbl> <dbl> <dbl> <int> <chr> <chr>
1 -0.686 0.297 0 -0.103 10 Overall Pre
2 -0.988 0.371 0 -0.261 10 Overall Post
3 -0.713 0.397 0 0.0657 10 Bulgaria Pre
4 -0.337 0.427 0 0.501 10 Finland Pre
5 -1.20 0.814 0 0.398 10 Germany Pre
6 -0.241 0.320 0 0.385 10 Bulgaria Post
7 -1.86 1.04 0 0.167 10 Finland Post
8 -2.63 1.30 0 -0.0907 10 Germany Post

# 9. Session info

sessionInfo()

R version 4.3.3 (2024-02-29 ucrt)
Platform: x86_64-w64-mingw32/x64 (64-bit)
Running under: Windows 11 x64 (build 22631)

Matrix products: default


locale:
[1] LC_COLLATE=English_United States.utf8
[2] LC_CTYPE=English_United States.utf8
[3] LC_MONETARY=English_United States.utf8
[4] LC_NUMERIC=C
[5] LC_TIME=English_United States.utf8

time zone: Europe/Sofia
tzcode source: internal

attached base packages:
[1] stats graphics grDevices utils datasets methods base

other attached packages:
 [1] posterior_1.5.0 tidybayes_3.0.6 brms_2.21.0 Rcpp_1.0.12
 [5] patchwork_1.2.0 ggdist_3.3.2 psych_2.4.3 broom_1.0.5
 [9] gtsummary_1.7.2 rstatix_0.7.2 purrr_1.0.2 stringr_1.5.1
[13] forcats_1.0.0 ggplot2_3.5.1 tidyr_1.3.1 dplyr_1.1.4

loaded via a namespace (and not attached):
 [1] mnormt_2.1.1 gridExtra_2.3 inline_0.3.19
 [4] sandwich_3.1-0 rlang_1.1.3 magrittr_2.0.3
 [7] multcomp_1.4-25 matrixStats_1.3.0 compiler_4.3.3
 [10] loo_2.7.0 reshape2_1.4.4 systemfonts_1.0.6
 [13] vctrs_0.6.5 httpcode_0.3.0 pkgconfig_2.0.3
 [16] arrayhelpers_1.1-0 crayon_1.5.2 fastmap_1.1.1
 [19] ellipsis_0.3.2 backports_1.4.1 labeling_0.4.3
 [22] utf8_1.2.4 promises_1.3.0 rmarkdown_2.26
 [25] ragg_1.3.0 xfun_0.43 jsonlite_1.8.8
 [28] later_1.3.2 uuid_1.2-0 parallel_4.3.3
 [31] R6_2.5.1 stringi_1.8.3 StanHeaders_2.32.7
 [34] car_3.1-2 estimability_1.5 rstan_2.32.6
 [37] knitr_1.46 zoo_1.8-12 bayesplot_1.11.1
 [40] httpuv_1.6.15 Matrix_1.6-5 splines_4.3.3
 [43] tidyselect_1.2.1 abind_1.4-5 yaml_2.3.8
 [46] codetools_0.2-19 curl_5.2.1 pkgbuild_1.4.4
 [49] plyr_1.8.9 lattice_0.22-5 tibble_3.2.1
 [52] shiny_1.8.1.1 withr_3.0.0 bridgesampling_1.1-2
 [55] askpass_1.2.0 flextable_0.9.5 coda_0.19-4.1
 [58] evaluate_0.23 survival_3.8-3 RcppParallel_5.1.7
 [61] zip_2.3.1 xml2_1.3.6 pillar_1.9.0
 [64] carData_3.0-5 tensorA_0.36.2.1 checkmate_2.3.1
 [67] stats4_4.3.3 distributional_0.4.0 generics_0.1.3
 [70] rstantools_2.4.0 munsell_0.5.1 scales_1.3.0
 [73] xtable_1.8-4 glue_1.7.0 gdtools_0.3.7
 [76] emmeans_1.10.1 tools_4.3.3 gfonts_0.2.0
 [79] data.table_1.17.8 mvtnorm_1.2-4 grid_4.3.3
 [82] QuickJSR_1.1.3 colorspace_2.1-0 nlme_3.1-164
 [85] cli_3.6.2 textshaping_0.3.7 officer_0.6.5
 [88] fontBitstreamVera_0.1.1 fansi_1.0.6 svUnit_1.0.6
 [91] broom.helpers_1.15.0 Brobdingnag_1.2-9 gt_0.10.1
 [94] V8_4.4.2 gtable_0.3.5 digest_0.6.35
 [97] fontquiver_0.2.1 crul_1.4.2 TH.data_1.1-2
[100] farver_2.1.1 htmltools_0.5.8.1 lifecycle_1.0.4
[103] mime_0.12 openssl_2.1.2 fontLiberation_0.1.0
[106] MASS_7.3-60.0.1
