# Supplementary material for: Virtual Reality–Enhanced Training for Trauma-Informed Care Among Residential and Child Mental Health Professionals: Pre-Post Evaluation Study
Source: JMIR Med Educ. 2026 Apr 17;12:e86543. doi: 10.2196/86543 (PMC13089627; doi:10.2196/86543)
Supplement: Checklist 2 [file mededu-v12-e86543-s004.docx]

TiDieR Checklist (Template for Intervention Description and Replication) (Hoffman et al., 2014)

| BRIEF NAME OF INTERVENTION | Safe4Child—Trauma-informed approaches to support children and adolescents experiencing distress. |
| --- | --- |
| WHY | Staff in residential child and adolescent services need practical, trauma-informed skills to reduce restrictive practices and respond safely to escalating distress. The program aims to introduce a trauma-sensitive lens that seeks the “good reasons” behind behavior; strengthen the therapeutic environment and therapeutic use of self; build de-escalation competence; and embed strategies that enhance safety and wellbeing for young people and professionals. Skill development is accelerated through an immersive VR simulation that turns abstract principles into hands-on practice. |
| WHAT  MATERIALS & PROCEDURES WERE USED? | **Materials**: five modules—four asynchronous online modules hosted on a learning management system and one in-person VR simulation.   - Module 1: Infant, child, and adolescent mental health. - Module 2: The therapeutic environment and the therapeutic use of self. - Module 3: Ethical, legislative, and trauma-informed perspectives on restrictive practice, restraint, and seclusion. - Module 4: Trauma-informed interventions for behaviors that challenge. - Module 5: VR simulation of a residential-care scenario involving a distressed child and standardized staff responses.   **Supporting materials** include brief video segments, readings, reflective prompts, 10-item self-check quizzes per module, continuous assessments, and course evaluation forms.  **Procedures**: learners receive guidance on pacing and assessment at program start. Modules 1–4 combine theory, media, self-reflection, and independent reading (approximately 25 hours per module). Each module concludes with a brief 10-question quiz to self-monitor progress before advancing. The final module is a facilitated, face-to-face VR session followed by a structured debrief to consolidate perceptions, emotions, and learning from the scenario. |
| WHO PROVIDED?  (Providers) | Academic staff and trained facilitators from four European universities: Hamburg University of Applied Sciences (Germany), Turku University of Applied Sciences (Finland), Medical University of Plovdiv (Bulgaria), and University College Cork (UCC, Ireland). |
| HOW IS IT DELIVERED? | Four asynchronous online modules followed by one standardized, in-person VR simulation with facilitator oversight and a guided debrief. |
| WHERE? | Online learning platform plus on-site VR delivery at the four partner universities. Training was implemented across all four sites; however, matched pre–post outcome assessment was completed in three countries (Germany, Finland, Bulgaria). The Irish site (UCC) delivered the training but did not contribute post-intervention assessments within the study window. |
| WHEN and HOW MUCH? | Four self-directed online modules over four weeks (approximately 25 hours per module), followed by a 2–3 hour in-person VR session and debrief. |
| TAILORING AND MODIFICATIONS | To address learner queries and technology/navigation issues, some sites added optional weekly synchronous support during the four asynchronous modules. Core content, VR scenario, and facilitation scripts were kept uniform across sites. |
| HOW WELL? | Facilitators followed a standardized script and safety protocol; VR sessions were mirrored to a monitor to allow real-time supervision and debriefing. Learner engagement was monitored through quiz completion and module progression logs. The program was well received. In the analytic sample with matched data (n = 79 across three countries), ARTIC-10 scores improved overall following the intervention. |

Hoffmann TC, Glasziou PP, Boutron I, et al. Better reporting of interventions: Template for intervention description and replication (TIDieR) checklist and guide. BMJ. Mar 7, 2014;348:g1687. [doi: 10.1136/bmj.g1687] [Medline: 24609605]
